# Efficacy of lumbar and abdominal muscle rehabilitation training on degree of osteoporosis, pain and anxiety in elderly patients with osteoporotic vertebral compression fracture after PKP and compliance analysis

**DOI:** 10.3389/fmed.2024.1364497

**Published:** 2024-06-28

**Authors:** Yaqin Xu, Dong Li, Qian Zhang, Lulu Tong

**Affiliations:** ^1^Department of Geriatric VIP No. 5 (Department of Geriatric Medicine), Zhejiang Provincial People’s Hospital, Affiliated People’s Hospital, Hangzhou Medical College, Health Management Center, Hangzhou, China; ^2^Department of Acupuncture No. 2, Zhejiang Provincial People’s Hospital, Affiliated People’s Hospital, Hangzhou Medical College, Health Management Center, Hangzhou, China; ^3^Department of Rehabilitation Medicine, Zhejiang Provincial People’s Hospital, Affiliated People’s Hospital, Hangzhou Medical College, Rehabilitation Medicine Center, Rehabilitation & Sports Medicine Research Institute of Zhejiang Province, Hangzhou, China

**Keywords:** osteoporotic vertebral compression fracture, percutaneous vertebroplasty with balloon dilatation, rehabilitation training, compliance, PKP

## Abstract

**Purpose:**

To explore the rehabilitation effect and compliance of lumbar and abdominal muscle rehabilitation training in patients with osteoporotic vertebral compression fracture (OVCF) after percutaneous balloon vertebroplasty (PKP).

**Methods:**

A total 177 elderly patients with OVCF were divided into rehabilitation group (*n* = 104) and control group (*n* = 73) according to whether they received psoas and abdominal muscle rehabilitation training for 3 months after PKP. The differences of general data, orthopaedic rehabilitation, prognosis and bone metabolism were compared between the two groups. All the patients were divided into compliance group (68 cases) and non-compliance group (36 cases) according to compliance. Orthopaedic rehabilitation indicators, prognostic indicators of PKP, and bone metabolism-related parameters were collected for analysis of Chi-square test and Logistic regression. ROC curve was used to analyze the predictive value of bone metabolism related indicators in the compliance of lumbar and abdominal muscle rehabilitation training.

**Results:**

There was no significant difference in the general data between the rehabilitation training group and the control group (All *p* > 0.05). Compared with the control group, the Berg balance scale score was significantly increased, while the Visual Analogue Scale (VAS) score, Oswestry Disability Index (ODI) score and the proportion of new fractures were significantly decreased in the rehabilitation training group (All *p* < 0.05). Compared with the control group, the bone mineral density (BMD) T value, osteocalcin (OCN) and 25-hydroxyvitamin D (25 (OH) D) levels were significantly increased and the levels of type I N-propeptide (P1NP) and β-isomerized C-terminal telopeptides (β-CTX) were significantly decreased in the rehabilitation training group compared with the control group (All *p* < 0.05). Chi-square test and Logistic regression analysis showed that age > 75 years, severe anxiety, severe pain and postoperative complications were significantly associated with the compliance of psoas and abdominal muscle rehabilitation training in patients with OVCF after PKP. ROC curve analysis showed that BMD T value, OCN, P1NP, β-CTX, or 25-OH-D levels predicted the AUC of rehabilitation training compliance in patients with OVCF after PKP were 0.821, 0.835, 0.736, 0.715, and 0.748, respectively.

**Conclusion:**

Rehabilitation training of lumbar and abdominal muscles can significantly improve the efficacy of PKP, reduce the degree of osteoporosis and improve the prognosis of patients with OVCF. Age, anxiety, pain and postoperative complications were independent risk factors affecting the compliance of psoas and abdominal rehabilitation training in patients with OVCF after PKP.

## 1 Introduction

Osteoporosis is a systemic disorder of bone metabolism characterized by damage of bone tissue microstructure, decreasing proportion of bone mineral composition and bone matrix, and increased bone fragility (1). With the aggravation of population aging, osteoporotic fracture has become one of the main diseases endangering the health of the elderly, leading to a significant decrease in the quality of life of patients (2). Osteoporotic fractures are usually caused by low-energy or nonviolent injuries and are a serious consequence of osteoporosis (2). Among them, osteoporotic vertebral compression fractures (OVCF) are common types of spinal fractures. The incidence of OVCF is generally higher in patients with osteoporosis. Improper treatment can easily cause severe deformation of the spine, which affects spinal function. Patients generally see a doctor with severe pain in the waist and mobility disturbance (3). Without prompt treatment, the progression of fracture collapse can lead to kyphosis, and chronic persistent pain will seriously affect the quality of life of the elderly (4).

Patients with OVCF generally have poor surgical tolerance due to advanced age and multiple underlying diseases (5, 6). Current treatment modalities for elderly patients with OVCF include conservative treatment, open surgery and minimally invasive surgery (4, 7, 8). Conservative treatment mainly includes bed rest (for at least 3 months), traditional reduction methods (e.g., manual reduction, traction reduction), external fixation of braces, and functional exercise, which are mainly applicable to those with mild symptoms or difficult to undergo surgical treatment (9). However, long-term bed rest is not only accompanied by an exacerbation of osteoporosis, but also a greatly increased risk of bedridden complications (e.g., hypostatic pneumonia, bedsore, venous thrombosis of lower extremities, etc.) (9). In addition, open surgical treatment has the disadvantages of high risk, large incision and large amount of bleeding, which leads to a long recovery time after open fracture surgery (10). Therefore, the main treatment modality for elderly patients with OVCF is minimally invasive surgery (11). Percutaneous kyphoplasty (PKP) is a minimally invasive procedure performed under C-arm fluoroscopy (12). The surgical incision is only about 0.5 cm, with the advantages of less bleeding, short operation time and rapid postoperative pain relief (12). In addition, early recovery of activity after PKP is the preferred treatment option for patients with OVCF in recent years.

Patients with osteoporosis tend to have balance dysfunction and a higher risk of falls (13). Of these, more than half of patients with OVCF still experience a second fall leading to a secondary fracture (13). Although PKP can provide some degree of reduction and fixation for collapsed vertebral bodies, most patients still have soft tissue injury and partial kyphosis after surgery (13). These factors are prone to decrease spinal structural stability and bias in the center of gravity, ultimately leading to pain, poor mobility and falls in patients with OVCF (14). Early postoperative rehabilitation exercise, as an important part of rehabilitation therapy, has become a routine treatment item after PKP (15–17). Early postoperative rehabilitation training based on strength training principles can improve postural control and muscle strength, increase postural stability, improve balance ability, relieve pain, and significantly improve patients’ activities of daily living (18). However, the elder patient may exhibit the lower acceptance for lumbar and abdominal muscle rehabilitation training, and difficult to complete the daily arrangement of lumbar and abdominal muscle rehabilitation training. In addition, the higher the degree of postoperative pain, the lower the acceptance of lumbar and abdominal muscle rehabilitation training. Therefore, there were large differences in compliance with early postoperative rehabilitation exercises. In order to improve the compliance of patients and improve the effectiveness of rehabilitation exercise, the purpose of this study was to investigate the effect of psoas and abdominal muscle rehabilitation training on the prognosis of patients with OVCF after PKP and the influencing factors affecting the compliance of rehabilitation exercise. Meanwhile, the effect of lumbar and abdominal muscle rehabilitation training on the rehabilitation of OVCF patients after PKP were also analyzed by different evaluation scales.

## 2 Data and methods

### 2.1 Clinical data

A total of 177 patients with OVCF who visited Zhejiang Provincial People’s Hospital from January 2022 to August 2023 were retrospectively collected. All patients with OVCF were divided into rehabilitation group (104 cases) and control group (73 cases) according to whether they received lumbar and abdominal muscle rehabilitation training. In addition, patients receiving psoas and abdominal muscle rehabilitation training were further divided into compliance group (68 patients) and noncompliance group (36 patients) according to compliance.

Inclusion criteria: (1) OVCF was diagnosed by X-ray, CT or MRI; (2) Received PKP treatment; (3) Age ≥ 60 years old; (4) Non-violent or low-energy injury was the causative factor.

Exclusion criteria: (1) Patients with incomplete clinical data; (2) Those who cannot cooperate with rehabilitation training in the whole course; (3) Patients with mental illness; (4) Patients with malignant tumors; (5) Patients with relevant diseases that have an impact on bone metabolism. The research related to human use has been complied with all the relevant national regulations, institutional policies and in accordance with the tenets of the Helsinki Declaration, and has been approved by the ethnical committee of Zhejiang Provincial People’s Hospital.

### 2.2 Treatment regimen

All patients received 3 months of lumbar and abdominal muscle rehabilitation training after PKP. Rest in supine position 8 h after PKP. From the first postoperative day, patients in both groups were able to start walking with waistlines. The psoabdominal muscle function training was started on the first day after PKP, and the training was continued for 3 months. Routine incision dressing change was performed after operation, and the anterolateral radiographs of thoracolumbar spine were reexamined. Depending on the time of discharge, waist circumference was continued until 3 months after discharge.

Self-management of rehabilitation was arranged by patients according to training before and after discharge. In addition, the physician regularly followed up the patients every week and recorded the completion of rehabilitation training for the patients. Lumboabdominal muscle rehabilitation training methods are as follows:

1. Lumbodorsal muscle training: the patient lies on the bed top, take the supine position, place the pillow back, both elbow and both soles on the bed surface as the supporting point, so that the axis of the lower leg is as 90° as possible from the bed surface, and naturally separated as wide as the shoulder. Lumbodorsal muscle strength mainly caused the two parts of the back and buttocks to leave the bed slowly, and the abdomen and knee were kept at the same level as much as possible. After the movement was in place for 5 s, then slowly placed on the bed. The rest interval between the two actions was 5 s, 20 times/group, and completed 5 groups per day (total of 100).
2. Abdominal muscle training 1: the patient lies on the bed, takes the supine position, puts his hands crossed over the abdomen, slowly contracts the abdominal muscles when exhales, feels the internal abdominal muscles slowly close to the spine, maintains the action for 20 s, then relaxes the muscles, and then relaxes the muscles one time. The rest interval of the two movements was 5 s, 20 times/group, and 5 groups (100 in total) were completed daily.
3. Abdominal muscle training 2: lie on the bed, take the supine position, hold the shoulders with both hands, then flex the hips and knees 90°, with abdominal muscle strength as the main starting point, so that the neck above the neck is slowly lifted out of the bed surface about 15 cm, conscious abdominal tighten, keep it in this position for 5 s, then slowly put it slowly to the bedside, and relax the whole body. The two movements were rested at an interval of 5 s, 20 times/group, and 5 groups (100 in total) were completed daily.

### 2.3 Observation measure

#### 2.3.1 Baseline data

Data including smoking history, alcohol consumption history, fracture site, fracture course, previous fracture history, and whether falls occurred in the past month were collected after admission.

#### 2.3.2 Relevant indicators of orthopaedic rehabilitation

All patients received Berg Balance Scale, Visual Analogue Scale (VAS) and Oswestry Disability Index (ODI) Scale at 3 months after surgery. At the same time, the occurrence of falls and clinical efficacy were statistically analyzed. Berg balance scale score: the balance function was assessed with Berg balance scale. The total score of Berg balance scale was 56. The higher the score indicated that the better the balance function. VAS: draw a straight line 10 cm long on the paper surface, and the patient marks the corresponding points on the line according to their current pain perception. The physician scored the location. The scores ranged from 0 to 10, and 0 to 4, 4 to 7, and 7 to 10 according to no/mild, moderate, and severe pain, respectively. The higher the score indicated the more severe the pain. ODI: a total of 10 questions were included, including the degree of low back pain and leg pain, personal self-care, weight lifting, walking, sitting, standing, sleep, sexual, social and travel. Each topic was subdivided into 5 grades, with higher scores indicating more pronounced dysfunction. All rating scale results were collected in a double-blind fashion by a physician.

Criteria for judging clinical efficacy: (1) Effective: fracture healing, basically no occurrence of low back pain, patients basically resumed daily life; (2) Ineffective: there was still some pain, unfavorable lumbar activity, no obvious improvement or even aggravation of the condition.

#### 2.3.3 Prognostic indicators of PKP

During the follow-up period of lumbar and abdominal muscle rehabilitation training, the new fracture of adjacent vertebral body, whether the patient needed further anti-osteoporosis treatment, and the situation of bone cement leakage were counted. New fractures were defined as nonoperative vertebral fractures occurring within 3 months of surgery. The patient’s cement leakage was assessed by CT. Leakage was judged if the cement exceeded the vertebral boundary.

#### 2.3.4 Bone metabolism-related parameters

Bone mineral density (BMD) T value was measured using GE Lunar-DPX-MD DXA. The T value of BMD of L1-L4 vertebrae in each patient was measured in anteroposterior position. According to WHO diagnostic criteria, T value> −1.0 SD was normal, −2.5 SD < T value≤ −1.0 SD was osteopenia, and bone mineral density T value≤ − 2.5 SD was osteoporosis. Serum osteocalcin levels (OCN) were measured by ELISA. The bone metabolism indexes including procollagen of type I N-propeptide (P1NP), β-isomerized C-terminal telopeptides (β-CTX), 25-hydroxyvitamin D (25-OH-D) and parathyroid hormone (PTH) were measured by Swiss Roche electrochemiluminescence analyzer Cobas e602.

#### 2.3.5 Compliance-related indicators

The early rehabilitation training compliance evaluation form was used to evaluate the compliance of rehabilitation training at 3 months after surgery. The contents of the evaluation form included 3 items, including the degree of knowledge of rehabilitation, the mastery of psoas and abdominal muscle exercise methods, and whether to complete the functional exercise program according to the guidance of medical staff. Compliance: Patients can take the initiative to complete the early functional exercise program developed by medical staff with high quality every day; Non-compliance: patients occasionally carry out rehabilitation training or do not follow the guidance of medical staff or refuse to carry out lumbar and abdominal muscle rehabilitation exercise. Lumbar and abdominal muscle rehabilitation training program for patients after PKP and patient compliance evaluation scale were referred to Supplementary Tables S1, S2. Self-rating Anxiety Scale (SAS) was used to evaluate the negative emotions of the patients.50–59 points were classified as no or mild anxiety, 60–69 as moderate anxiety, and ≥ 70 as severe anxiety. Higher scores represent more severe anxiety. Glasgow Soma Scale (GCS) was used to assess the degree of consciousness disturbance. GCS totaled 15 points, 11 to 15 as no or mild disturbance of consciousness, 6 to 10 as moderate disturbance of consciousness, and 0 to 5 as severe disturbance of consciousness. The lower the score, the worse the patient’s state of consciousness.

### 2.4 Statistical analysis

Statistical analysis was performed using SPSS 27.0 soft. Measurements that meet the normal distribution are expressed as mean ± SD, and the inter-group comparison is performed using an independent sample T-test. Measurements that did not meet the normal distribution were expressed as median (quartile), and nonparametric rank sum test was used for group comparisons. Enumeration data were expressed as rate (%) or constituent ratio, and chi-square test was used for comparison between groups. Binary Logistic regression was used for influencing factor analysis. *p* < 0.05 was statistically significant.

## 3 Results

### 3.1 Analysis of general data of OVCF patients

According to the inclusion criteria and exclusion criteria, 177 patients with OVCF who underwent PKP were included in this study. According to whether they received lumbar and abdominal muscle rehabilitation training, all patients were divided into rehabilitation training group (104 cases) and control group (73 cases). The patients in the rehabilitation training group received postoperative anti-osteoporosis treatment and rehabilitation training, while the control group received only postoperative anti-osteoporosis treatment. There were no dropouts after 3 months of follow-up. Baseline data for patients with OVCF are presented in Table 1. Compared with the control group, there was no statistically significant difference in sex composition, age, height, weight, diabetes, hypertension, previous regular exercise, smoking history, alcohol consumption history, fracture site, disease course, previous fracture history, and the number of patients with falls in the past month between the rehabilitation training group (All *p* > 0.05).

**Table 1:** Analysis of general data of OVCF patients.

Parameters	Rehabilitation training group (*n* = 104)	Control group (*n* = 73)	t/Z/*X*^2^	*p* value
Gender (male/female)	48/56	34/39	0.003	0.956
Age	71 (67, 77)	71 (68, 75)	−0.428	0.669
Height (cm)	162.71 ± 6.64	163.63 ± 6.94	−0.889	0.375
Weight (kg)	57.78 ± 9.31	59.82 ± 6.11	−1.763	0.080
Diabetes	4 (3.85%)	2 (2.74%)	0.160	0.689
Hypertension	27 (25.96%)	15 (20.55%)	0.695	0.405
Previous regular exercise	11 (10.58%)	8 (10.96%)	0.007	0.936
Smoking history	43 (41.35%)	34 (46.58%)	0.477	0.490
Drinking history	27 (25.96%)	15 (20.55%)	0.695	0.405
Fracture site (thoracic/lumbar)	61/43	45/28	0.160	0.690
Illness course (days)	6 (5, 6)	5 (5, 6)	−0.867	0.386
Fracture history	52 (50.00%)	36 (49.32%)	0.008	0.929
Fall in the past month	49 (47.12%)	30 (41.10%)	0.629	0.428

### 3.2 Effect of lumboabdominal muscle rehabilitation training on postoperative efficacy of PKP in patients with OVCF

The efficacy of psoabdominal muscle rehabilitation training in patients with OVCF after PKP is shown in Table 2. The score of Berg balance scale in the rehabilitation training group was significantly higher than that in the control group (*p* < 0.001), indicating that the balance function was significantly improved in patients with OVCF after lumbar and abdominal muscle rehabilitation training. In addition, the VAS and ODI scores in the rehabilitation training group were significantly lower than those in the control group (Both *p* < 0.001), suggesting that the pain degree and self-care ability of the rehabilitation training group were significantly improved. The frequency of falls in patients with OVCF within 3 months after PKP was statistically analyzed. The results showed that the frequency of falls in the rehabilitation training group was significantly lower than that in the control group (*p* < 0.05). In addition, the overall clinical efficacy of OVCF patients after 3 months of lumbar and abdominal muscle rehabilitation training was significantly better than that of the control group (*p* < 0.001). In summary, lumbar and abdominal muscle rehabilitation training can significantly improve the clinical efficacy of PKP in patients with OVCF.

**Table 2:** Comparison of orthopaedic rehabilitation-related indicators in different groups of OVCF patients.

Parameters	Rehabilitation training group (*n* = 104)	Control group (*n* = 73)	Z/*X*^2^	*p* value
Berg balance scale	48.50 (46.18, 50.26)	28.32 (26.00, 30.85)	−11.312	<0.001
VAS	2.81 (2.29, 3.55)	4.12 (2.81, 5.62)	−4.269	<0.001
ODI	17.46 (16.40, 18.29)	20.54 (19.48, 21.94)	−9.664	<0.001
Postoperative fall	4 (3.85%)	10 (13.70%)	5.716	0.017
Clinical efficacy (effective/ineffective)	98/6	54/19	14.513	<0.001

### 3.3 Effect of lumboabdominal muscle rehabilitation training on prognosis of patients with OVCF

To further analyze the effect of lumbar and abdominal muscle rehabilitation training on the prognosis of patients with OVCF after PKP. As shown in Table 3, only 19 (18.27%) or 15 (14.42%) patients in the rehabilitation group experienced fracture or cement leakage after PKP compared with 23 (31.51%) or 19 (26.03%) patients in the control group, respectively. Chi-square test showed that the proportion of new fractures in the rehabilitation training group was significantly lower than that in the control group (*p* < 0.05), but there was no significant difference in the proportion of bone cement leakage (*p* > 0.05). In addition, 64 patients (61.54%) in the rehabilitation group and 61 patients (83.56%) in the control group still needed further anti-osteoporosis treatment after 3 months of psoas and abdominal muscle rehabilitation training. Chi-square test showed that the proportion of patients who still needed anti-osteoporosis treatment in the rehabilitation training group was significantly lower than that in the control group (*p* < 0.01). The above results showed that psoas and abdominal muscle rehabilitation training could significantly improve the prognosis of patients with OVCF after PKP.

**Table 3:** Comparison of prognosis in patients with different OVCF.

Parameters		Rehabilitation training group (*n* = 104)	Control group (*n* = 73)	X^2^	*p* value
New fracture	Yes	19	23	4.153	0.042
	No	85	50		
Cement leakage	Yes	15	19	3.722	0.054
	No	89	54		
Anti-osteoporosis therapy	Yes	64	61	10.027	0.002
	No	40	12		

### 3.4 Effect of lumboabdominal muscle rehabilitation training on osteoporosis-related indexes in OVCF patients

The degree of osteoporosis in patients with OVCF is an important factor to evaluate the efficacy of PKP. Therefore, we further evaluated the effect of lumbar and abdominal muscle rehabilitation training on osteoporosis-related indicators in patients with OVCF. As shown in Table 4, the BMD T value of the rehabilitation training group was significantly higher than that of the control group (*p* < 0.001), indicating that the overall degree of osteoporosis was significantly improved after OVCF rehabilitation training via the psoas and abdominal muscles. In addition, both OCN and PTH in the rehabilitation group were significantly higher than those in the control group (Both *p* < 0.05). In contrast, Both P1NP and beta-CTX were significantly lower in the rehabilitation group than in the control group (Both *p* < 0.001). The above results showed that the lumbar and abdominal muscle rehabilitation training could significantly improve the degree of osteoporosis in patients with OVCF after PKP.

**Table 4:** Comparison of osteoporosis-related indicators in different OVCF patients.

Parameters	Rehabilitation training group (*n* = 104)	Control group (*n* = 73)	*t* value	*p* value
BMD T value	−2.41 ± 0.55	−3.12 ± 0.48	8.872	<0.001
OCN (μg/L)	5.59 ± 1.27	5.20 ± 1.33	1.987	0.048
P1NP (μg/L)	41.76 ± 8.06	59.00 ± 10.47	−11.829	<0.001
β-CTX (ng/L)	478.39 ± 75.74	594.81 ± 77.79	−9.955	<0.001
25(OH)D (μg/L)	15.12 ± 4.02	9.86 ± 2.79	10.281	<0.001
PTH (ng/L)	71.10 ± 19.82	56.57 ± 15.90	5.198	<0.001

### 3.5 Analysis of influencing factors of lumboabdominal muscle rehabilitation training compliance in OVCF patients

The compliance of 3 months after PKP was evaluated according to the compliance evaluation form of early rehabilitation training and divided into compliance group (68cases) and non-compliance group (36 cases). The analysis of factors influencing compliance with lumbar and abdominal muscle rehabilitation training in patients with OVCF after PKP is shown in Table 5. There were 34 males and 34 females in the compliance group, while 14 males and 22 females in the noncompliance group, the difference was not statistically significant (*p* > 0.05). In addition, the proportion of patients>75 years of age was 23.53% in the compliance group, which was significantly lower than that in the noncompliant group (44.44%) (*p* < 0.05), suggesting that the older the age, the higher the frequency of noncompliance. Moreover, the postoperative anxiety, postoperative pain and the frequency of osteoporosis in the compliance group were significantly lower than those in the non-compliance group (All *p* < 0.05). However, there was no significant difference in the frequency of postoperative disturbance of consciousness between the two groups (*p* > 0.05). In terms of the prognosis of PKP, the proportion of patients requiring further anti-osteoporotic treatment and the proportion of patients with cement leakage were significantly lower in the compliance group than in the non-compliance group (Both *p* < 0.05), while the proportion of patients with new fractures was not statistically different between the two groups (*p* > 0.05).

**Table 5:** Univariate analysis of compliance with lumbar and abdominal muscle rehabilitation training in patients with OVCF.

Parameters		Compliance group (*n* = 68)	Non-compliant group (*n* = 36)	X^2^	*p* value
Age	>75	16	16	4.834	0.028
	≤75	52	20		
Gender	Male	34	14	1.169	0.280
	Female	34	22		
Postoperative anxiety	No or mild	9	1	7.236	0.027
	Moderate	52	25		
	Severe	7	10		
Postoperative disturbance of consciousness	No or mild	52	21	3.713	0.156
	Moderate	12	11		
	Severe	4	4		
Degree of postoperative pain	No or mild	59	24	8.446	0.015
	Moderate	8	7		
	Severe	1	5		
Degree of osteoporosis	Osteopenia	47	15	7.367	0.007
	Osteoporosis	21	21		
New fracture	Yes	9	10	3.334	0.068
	No	59	26		
Anti-osteoporosis therapy	Yes	37	27	4.215	0.040
	No	31	9		
Cement leakage	Yes	6	9	4.990	0.025
	No	62	27		

### 3.6 Logistic regression analysis of lumboabdominal muscle rehabilitation training compliance in patients with OVCF

**Figure 1:**
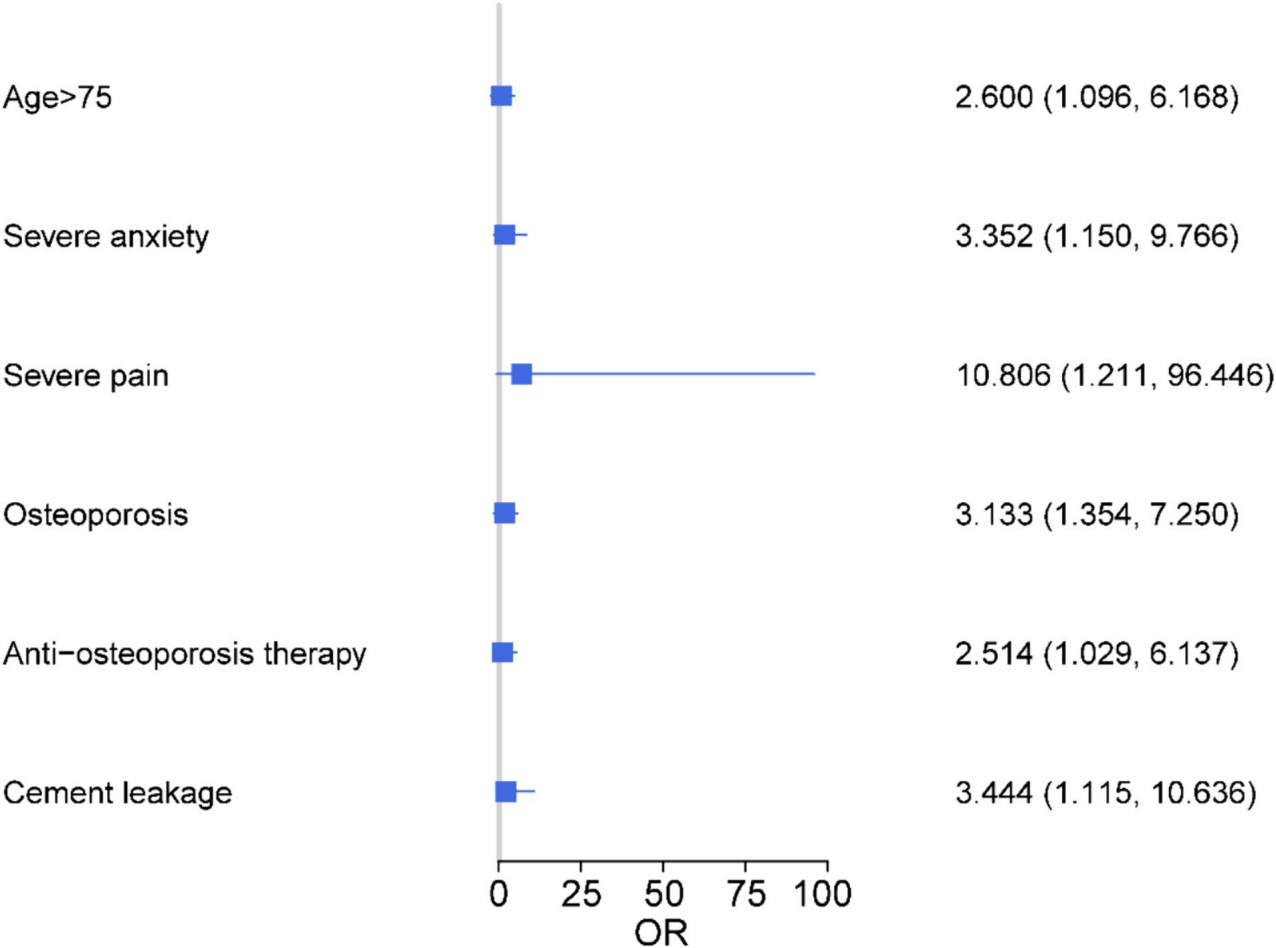
Analysis of influencing factors for compliance of OVCF patients after PKP surgery.

Logistic regression analysis was performed with patient compliance as dependent variable and factors with statistically significant differences in univariate analysis as independent variables, and the results are shown in Figure 1 and Table 6. Age > 75 years, postoperative complications, severe postoperative anxiety, severe postoperative pain, osteoporosis, anti-osteoporosis treatment and bone cement leakage were independent risk factors for poor compliance in patients with OVCF after PKP (All *p* < 0.05), and the probability of noncompliance increased 1.600, 1.292, 2.352, 9.806, 1.514 and 2.444 times, respectively.

**Table 6:** Logistic regression analysis of lumboabdominal muscle rehabilitation training compliance in patients with OVCF.

Parameters	β	S.E	Wald	*p*	OR	95%CI
Age > 75	0.956	0.441	4.701	0.030	2.600	1.096 ~ 6.168
Severe postoperative anxiety	1.089	0.550	3.926	0.027	3.352	1.150 ~ 9.766
Severe postoperative pain	1.743	1.212	2.070	0.033	10.806	1.211 ~ 96.446
Osteoporosis	1.142	0.428	7.121	0.008	3.133	1.354 ~ 7.250
Anti-osteoporosis therapy	0.922	0.455	4.095	0.043	2.514	1.029 ~ 6.137
Cement leakage	1.237	0.575	4.622	0.032	3.444	1.115 ~ 10.636

### 3.7 Predictive efficacy of serum bone metabolism indicators on the compliance of rehabilitation training in OVCF patients

**Figure 2:**
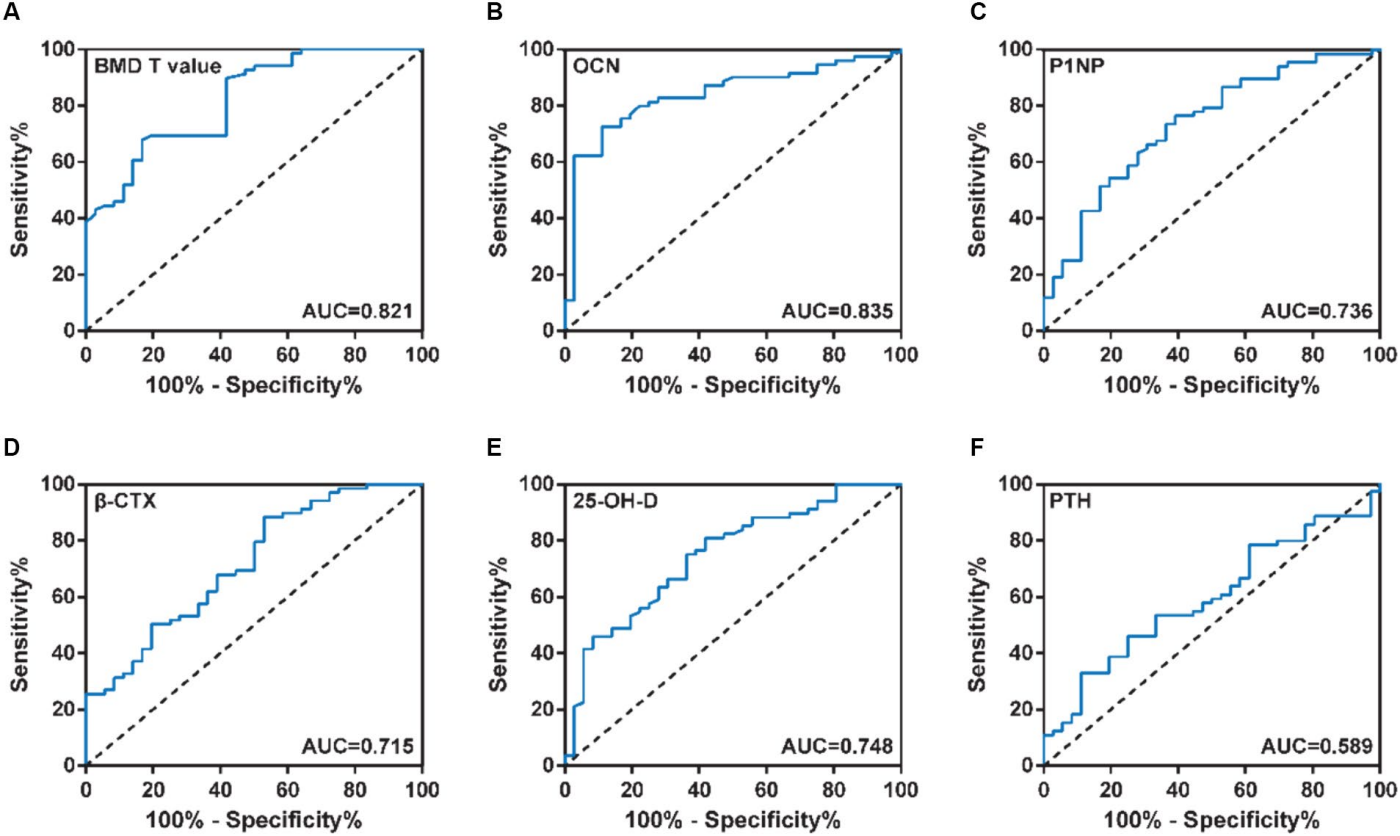
**(A)** BMD T value, **(B)** OCN, **(C)** P1NP, **(D)** β-CTX, **(E)** 25-OH-D and **(F)** PTH levels predicted rehabilitation training compliance in patients with OVCF after PKP for AUC.

The occurrence of osteoporosis after PKP is one of the important factors affecting patient compliance. Therefore, we analyzed the predictive efficacy of different serum bone metabolism-related indicators on rehabilitation training compliance in patients with OVCF after PKP by ROC curves. As shown in Figure 2 and Table 7, BMD T value, OCN, P1NP, β-CTX, or 25-OH-D levels predicted rehabilitation training compliance in patients with OVCF after PKP for AUC greater than 0.7, suggesting that these serum bone metabolism parameters have high predictive power. Of these, OCN had the highest AUC of 0.835. When the cut-off value was 5.44 μg/L, the sensitivity and specificity of OCN in rehabilitation training compliance were 72.1 and 88.9%, respectively. However, the AUC at PTH level was only 0.589 and was not statistically different (*p* > 0.05). These results suggest that BMD T value, OCN, P1NP, β-CTX or 25-OH-D levels can be used as predictors of rehabilitation compliance in patients with OVCF after PKP.

**Table 7:** Diagnostic efficacy of serum bone metabolic indicators for compliance with rehabilitation training in patients with OVCF.

Parameters	AUC (95% CI)	Cut-off	Sensitivity	Specificity	*p* value
BMD T value	0.821 (0.739, 0.902)	−2.35	67.6%	83.3%	<0.001
OCN	0.835 (0.754, 0.915)	5.44 μg/L	72.1%	88.9%	<0.001
P1NP	0.736 (0.636, 0.837)	45.85 μg/L	76.5%	61.1%	<0.001
β-CTX	0.715 (0.612, 0.818)	527.40 ng/L	88.2%	47.2%	<0.001
25-OH-D	0.748 (0.650, 0.846)	13.16 μg/L	80.9%	58.3%	<0.001
PTH	0.589 (0.478, 0.700)	85.30 ng/L	32.4%	88.9%	0.136

## 4 Discussion

The bone quality of the elderly is poor, so the leading cause of osteoporotic fractures is usually non-violent or low-energy injury (19). Osteoporotic fractures are most common in the elderly, with an incidence of approximately 49% of osteoporotic fractures (20). Fractures tend to occur at the thoracolumbar junction, often manifesting as loss of vertebral height, involving the anterior and middle columns of the vertebral body (2). Currently, the main modalities of minimally invasive treatment of OVCF are percutaneous vertebroplasty (PVP) and percutaneous balloon-expandable vertebroplasty (PKP) (21). PKP is the development and extension of PVP, and the main points of operation between the two are mainly different from balloon dilatation resulting in endplate collapse and partial correction of vertebral Cobb angle (22). Studies have demonstrated that PKP has a lower incidence of cement leakage, reduced progression of Cobb angle, and is more durable in terms of pain relief than PVP (23). In addition, PKP has a wide range of indications, except for vertebral compression fractures, but also has therapeutic effects on invasive intraspinal hemangiomas or vertebral metastases (24).

PKP provides some degree of reduction and internal fixation for fracture. Functional exercise and anti-osteoporosis therapy are the direction of perioperative and even long-term treatment for OVCF. Because patients lack sufficient attention to dysfunction after PKP, very few patients will choose further rehabilitation, greatly increasing the risk of falls and secondary fractures (16). Functional exercise as early as possible after surgery can accelerate the recovery of muscle strength, avoid muscle atrophy, and promote fracture healing through the stress stimulation of the muscle on the skeleton (25). In addition, local blood circulation can be accelerated by muscle movement and edema absorption can be promoted (25). Moreover, rehabilitation exercise is of great significance to prevent bone loss, fall and fracture recurrence after operation, and is beneficial to the recovery of postoperative function and the improvement of quality of life. Elderly patients are characterized by a high risk of falls, nonviolent injury as a contributing factor, and a high risk of recurrence after fracture surgery (26). The trunk is the core of motor control. The level of trunk control determines walking ability (including walking speed, efficiency balance, etc.). Therefore, the prevention of falls in patients after PKP should be mainly based on the training of the function of the abdominal muscles of the lower back muscles. Therefore, the aim of this study was to investigate the efficacy of lumboabdominal muscle functional training in patients with OVCF after PKP. In addition, there was a large difference in compliance among patients with OVCF after PKP. Therefore, this study will further explore the factors influencing the compliance of patients with psoas and abdominal muscle rehabilitation training.

A total of 104 patients in the rehabilitation training group and 73 in the control group were included in this study. There was no statistically significant difference in sex composition, age, smoking history, alcohol consumption history, fracture site, disease course, previous fracture history, and the number of patients with falls in the past month between the two groups (All *p* > 0.05), and the overall data were comparable. Studies have shown that osteoporosis can cause low back pain and scoliosis, with poor posture control relative to normal subjects, increasing the risk of falls and leading to new vertebrae (26). Decreased lumbar and back muscle strength in patients with osteoporotic compression fractures can lead to sagittal kyphosis deformity of the patient’s spine. The magnitude of left–right sway at the center of gravity is significantly increased while walking, and the anterior–posterior movement distance is decreased (2). Patients with OVCF have a progressive decline in their ability to maintain body homeostasis, and abnormal gait is accompanied by an increased risk of falls and a significantly increased risk of fractures (2). Moreover, falls are the main predisposing factor leading to the occurrence of first fragility fractures and re-fractures in patients (27). In addition, it has been shown that the primary cause of falls is imbalance (28). This suggests that the prevention of falls is essential to reduce the risk of osteoporotic vertebral fractures and re-fractures in the elderly (27). In this study, patients had significant improvements in balance function, pain severity, and risk of falls after 3 months of psoas and abdominal function training. Moreover, psoas and abdominal muscle rehabilitation training further improved the prognosis of patients with OVCF after PKP compared with controls, mainly including reducing the incidence of postoperative re-fractures and cement leakage, and reducing the proportion of patients still requiring further anti-osteoporotic treatment.

The degree of osteoporosis is a major factor in the development of OVCF (29). Current screening for fracture risk and the diagnosis of osteoporosis are usually based on BMD results (30). However, radiographic measurements of bone mass are not sensitive, and the physiological changes of bone respond slowly and do not reflect the state of bone metabolism early (31). Therefore, BMD measurements alone do not adequately predict fracture risk early (31). Bone turnover markers are a series of breakdown products released by osteoblasts and osteoclasts during bone remodeling and reflect their activity, including bone resorption marker β-CTX, which evaluates osteoclast activity and bone formation markers OCN and P1NP reflecting osteoblast activity (32). These indicators are able to early judge bone metabolic status and predict the risk of osteoporotic fracture (33). In addition, serum 25 (OH) D is a commonly used indicator reflecting the nutritional status of vitamin D in humans and plays a promoting role in maintaining homeostasis of bone homeostasis and bone mineral content (34). In addition, PTH can act on osteoblasts and promote osteoclast maturation, increased bone resorption, etc. (35). Therefore, serum 25 (OH) D and PTH levels also reflect the state of bone metabolism to some extent. Fluctuations in bone metabolism indicators can affect the activity of osteocytes and osteoclasts through multiple pathways. The rate of bone formation lags behind bone loss, resulting in decreased bone quality, which then leads to osteoporosis, leading to an increased risk of fracture (36). In this study, psoas and abdominal muscle rehabilitation training significantly increased BMD T value, OCN, and PTH levels, while reducing P1NP and β-CTX levels. The above results showed that the lumbar and abdominal muscle rehabilitation training could significantly improve the degree of osteoporosis in patients with OVCF after PKP.

At present, there is no study on the compliance of early rehabilitation exercise after PKP. Therefore, we further evaluated compliance and its influencing factors in 104 patients undergoing lumboabdominal muscle rehabilitation training. Elderly patients have weak immunity, poor nutritional status, more underlying diseases and weak self-recovery capacity. The results showed that patients >75 years of age in the compliance group were significantly lower than those in the noncompliant group, suggesting a significant association between age and patient compliance. Logistic regression analysis showed that patients aged >75 years had a 1.6-fold higher probability of poor compliance with psoas and abdominal muscle rehabilitation training. The occurrence of postoperative complications will hinder or delay the occurrence of early rehabilitation exercise to some extent (37). In addition, the occurrence of complications will, to a certain extent, limit the early rehabilitation exercise of patients, increase the psychological and physiological burden of patients, further cause patients’ anxiety and reduce patient compliance (38). In this study, postoperative complications (new fractures, cement leakage) and the proportion of patients requiring further antiosteoporotic treatment were significantly lower in the compliance group than in the noncompliant group. In addition, patients with severe postoperative anxiety had a 2.352-fold increased probability of poor compliance. On the other hand, standardized pain management can effectively promote early postoperative rehabilitation exercises and promote the recovery of patients’ early function (39). Pain management is beneficial to reduce postoperative pain and reduce the burden of postoperative rehabilitation exercises (40). At the same time, good pain management is an important means to promote rehabilitation exercise (41). The results of this study showed that the degree of postoperative pain was significantly lower in the compliance group than in the noncompliant group. Furthermore, severe postoperative pain is the most important factor affecting patient compliance. The risk of non-compliance with lumbar and abdominal muscle rehabilitation training in patients with severe postoperative pain was significantly increased by 9.806-fold. In summary, age, postoperative anxiety, postoperative pain, and postoperative complications were independent risk factors affecting the compliance of lumbar and abdominal rehabilitation training in patients with OVCF after PKP. Therefore, it is still necessary to further develop a personalized program for lumbar and abdominal muscle rehabilitation training according to the patient’s condition.

The limitations of lumbar and abdominal muscle rehabilitation training are mainly reflected in compliance. The compliance of rehabilitation training refers to the degree to which the patient’s training behavior is consistent with the doctor’s order. Poor compliance can lead to failure of the treatment effect to meet the expected criteria, thereby accelerating the course of the disease, increasing the risk of complications, and thus affecting the patient’s prognosis. Therefore, early and accurate assessment of rehabilitation compliance and intervention of poor compliance in patients with OVCF after PKP is of great significance in promoting the effect of rehabilitation training of psoas and abdominal muscles and improving the prognosis of patients. At present, the compliance of patients after PKP can only be evaluated by subjective feelings of patients and compliance evaluation scales. Therefore, we further analyzed the predictive efficacy of different bone metabolism indices on the compliance of rehabilitation training patients, with the aim of assessing the degree of osteoporosis, and at the same time preliminarily predicting the compliance of patients with psoas and abdominal muscle rehabilitation training. The results showed that the AUCs of BMD T value, OCN, P1NP, β-CTX or 25-OH-D levels in predicting rehabilitation compliance in patients with OVCF after PKP were 0.821, 0.835, 0.736, 0.715 and 0.748, respectively, suggesting that these serum markers of bone metabolism have high predictive power. Among them, OCN had the highest predictive power, and the diagnostic sensitivity and specificity were 72.1 and 88.9%, respectively.

The study has the following limitations: (1) The sample size of this study is small, and the results may be biased; (2) All the included cases were from a single center, which limited the universality of the study results to some extent; (3) The compliance of the patients is derived from the subjective answers of the patients, while the patients tend to choose the positive choices expected to be achieved by their own behavior when answering the subjective questions, which may lead to the evaluation results different from their true level.

In conclusion, lumbar and abdominal muscle rehabilitation training in patients with OVCF can significantly improve the efficacy of PKP, reduce the degree of osteoporosis and improve the prognosis. In addition, age, anxiety, pain and postoperative complications were independent risk factors for compliance with psoas and abdominal rehabilitation training in patients with OVCF after PKP. In addition, bone metabolism can be used as an effective method to predict the compliance of psoas and abdominal muscle rehabilitation training in patients after PKP. In the future, the sample size and scope of the study can be expanded, and longitudinal studies can be conducted to further evaluate the predictive value of compliance assessment indicators of lumbar and abdominal muscle rehabilitation training in patients with OVCF after PKP, so as to better guide clinical work.

## Data availability statement

The original contributions presented in the study are included in the article/Supplementary material, further inquiries can be directed to the corresponding author.

## Ethics statement

The studies involving humans were approved by the ethical committee of Zhejiang Provincial People’s Hospital. The studies were conducted in accordance with the local legislation and institutional requirements. Written informed consent for participation in this study was provided by the participants’ legal guardians/next of kin.

## Author contributions

YX: Conceptualization, Data curation, Investigation, Writing – original draft, Writing – review & editing. DL: Formal analysis, Methodology, Validation, Writing – original draft, Writing – review & editing. QZ: Resources, Visualization, Writing – original draft, Writing – review & editing. LT: Project administration, Supervision, Writing – original draft, Writing – review & editing.
