# Supplementary material for: Efficacy of lumbar and abdominal muscle rehabilitation training on degree of osteoporosis, pain and anxiety in elderly patients with osteoporotic vertebral compression fracture after PKP and compliance analysis
Source: Front Med (Lausanne). 2024 Jun 28;11:1364497. doi: 10.3389/fmed.2024.1364497 (PMC11245735; doi:10.3389/fmed.2024.1364497)
Supplement: Supplementary file 1 [file Data_Sheet_1.docx]

**Table S1**. Lumbar and Abdominal Muscle Rehabilitation Training Program for Patients after PKP.

|  | Time point | Duration | Number of repetitions |
| --- | --- | --- | --- |
| Lumbodorsal muscle training | 9:00 am | 5 s | 20 repetitions |
| Abdominal muscle training 1 |  | 20 s |  |
| Abdominal muscle training 2 |  | 5 s |  |
| Lumbodorsal muscle training | 12:00 am | 5 s | 20 repetitions |
| Abdominal muscle training 1 |  | 20 s |  |
| Abdominal muscle training 2 |  | 5 s |  |
| Lumbodorsal muscle training | 3:00 pm | 5 s | 20 repetitions |
| Abdominal muscle training 1 |  | 20 s |  |
| Abdominal muscle training 2 |  | 5 s |  |
| Lumbodorsal muscle training | 6:00 pm | 5 s | 20 repetitions |
| Abdominal muscle training 1 |  | 20 s |  |
| Abdominal muscle training 2 |  | 5 s |  |
| Lumbodorsal muscle training | 9:00 pm | 5 s | 20 repetitions |
| Abdominal muscle training 1 |  | 20 s |  |
| Abdominal muscle training 2 |  | 5 s |  |

**Table S2**. Patient Compliance Evaluation Scale

| Item | Presentation | Score |
| --- | --- | --- |
| Understanding of rehabilitation training methods | Be able to accurately repeat the items and process of rehabilitation training | 1 |
|  | Unable to accurately repeat rehabilitation training program and process | 0 |
| Whether rehabilitation training is completed accurately according to the schedule | Accurately complete rehabilitation training according to schedule | 1 |
|  | Failure to complete rehabilitation training according to schedule | 0 |
| Whether rehabilitation training is completed daily | Yes | 1 |
|  | No | 0 |

Score = 3: Compliance; Score < 3: Non-compliance
